# Magnitude and trends in socio-economic and geographic inequality in access to birth by cesarean section in Tanzania: evidence from five rounds of Tanzania demographic and health surveys (1996–2015)

**DOI:** 10.1186/s13690-020-00466-3

**Published:** 2020-09-15

**Authors:** Gebretsadik Shibre, Betregiorgis Zegeye, Bright Opoku Ahinkorah, Mpho Keetile, Sanni Yaya

**Affiliations:** 1 Department of Reproductive, Family and Population Health, School of Public Health, Addis Ababa University, Addis Ababa, Ethiopia; 2 Shewarobit Field Office, HaSET Maternal and Child Health Research Program, Addis Ababa, Ethiopia; 3 School of Public Health, Faculty of Health, University of Technology Sydney, Sydney, NSW Australia; 4 Population Studies and Demography, University of Botswana, Gaborone, Botswana; 5 School of International Development and Global Studies, University of Ottawa, 120 University Private, Ottawa, Ontario K1N 6N5 Canada; 6 The George Institute for Global Health, Imperial College London, London, United Kingdom

**Keywords:** Caesarean section, Inequality, Global health, Tanzania, DHS

## Abstract

**Background:**

Majority of maternal deaths are avoidable through quality obstetric care such as Cesarean Section (CS). However, in low-and middle-income countries, many women are still dying due to lack of obstetric services. Tanzania is one of the African countries where maternal mortality is high. However, there is paucity of evidence related to the magnitude and trends of disparities in CS utilization in the country. This study examined both the magnitude and trends in socio-economic and geographic inequalities in access to birth by CS.

**Methods:**

Data were extracted from the Tanzania Demographic and Health Surveys (TDHSs) (1996–2015) and analyzed using the World Health Organization’s (WHO) Health Equity Assessment Toolkit (HEAT) software. First, access to birth by CS was disaggregated by four equity stratifiers: wealth index, education, residence and region. Second, we measured the inequality through summary measures, namely Difference (D), Ratio (R), Slope Index of Inequality (SII) and Relative Index of Inequality (RII). A 95% confidence interval was constructed for point estimates to measure statistical significance.

**Results:**

The results showed variations in access to birth by CS across socioeconomic, urban-rural and regional subgroups in Tanzania from 1996 to 2015. Among the poorest subgroups, there was a 1.38 percentage points increase in CS coverage between 1996 and 2015 whereas approximately 11 percentage points increase was found among the richest subgroups within same period of time. The coverage of CS increased by nearly 1 percentage point, 3 percentage points and 9 percentage points among non-educated, those who had primary education and secondary or higher education, respectively over the last 19 years. The increase in coverage among rural residents was 2 percentage points and nearly 8 percentage points among urban residents over the last 19 years. Substantial disparity in CS coverage was recorded in all the studied surveys. For instance, in the most recent survey, pro-rich (RII = 15.55, 95% UI; 10.44, 20.66, SII = 15.8, 95% UI; 13.70, 17.91), pro-educated (RII = 13.71, 95% UI; 9.04, 18.38, SII = 16.04, 95% UI; 13.58, 18.49), pro-urban (*R* = 3.18, 95% UI; 2.36, 3.99), and subnational (D = 16.25, 95% UI; 10.02, 22.48) absolute and relative inequalities were observed.

**Conclusion:**

The findings showed that over the last 19 years, women who were uneducated, poorest/poor, living in rural settings and from regions such as Zanzibar South, appeared to utilize CS services less in Tanzania. Therefore, such subpopulations need to be the central focus of policies and programmes implemmentation to improve CS services coverage and enhance equity-based CS services utilization.

## Background

In 2017, there was a decline in maternal mortality rate from 451,000 in 2000 to 295,000. Despite this decline, globally, more than 800 maternal deaths are recoded every day from pregnancy and childbirth-related complications. Moreover, 20 women worldwide suffer from serious injuries, infections or disabilities [1]. More than two-third of maternal deaths occur in sub-Saharan Africa (68%) per year globally. This figure corresponds to approximately 533 maternal deaths per 100,000 live births, or 200,000 maternal deaths a year [1].

Majority of maternal deaths can be avoided through the utilization of quality obstetric services. However, many women continue to die because of lack of access to life-saving services including caesarean section (CS) [2]. CS has been regarded as one of the benchmarks for assessing progress in emergency obstetric care, and a method to avert complications during labor and delivery [3, 4]. The significance of CS in resource-poor settings is difficult to describe. In many low- and middle-income countries, CS has been reportedly underutilized especially among the disadvantaged populations, while the privileged groups often have access to CS [5, 6]. CS has become a useful medium to curb the menace of maternal deaths through improvement in the quality and use of maternal services for the management and treatment of complications in pregnancy, labor and delivery [7].

Globally, the effects of inequitable access to health are quite surprising as the extent of morbidities and deaths emanating from resource-poor countries are significantly higher than those in resource-rich nations [8]. The disparity in access to health is even more visible when analyzed within each country, especially in the resource-constrained countries [2]. The steepness of this disparity calls for the need to assess the notion of equitable access to health care that governments uniformly claim to be implementing [9]. Generally, there is sufficient evidence on the link between inequitable access to health care and inequitable distribution of illness [10, 11]. Diminishing the wide gap in important health care services use between different population groups would increase health equity and improve the overall health of a nation.

Limited access to CS has been considered as a contributing factor to the increase in maternal and neonatal mortality [12, 13]. While women in the richest households are overusing CS due to its convenience compared to the pain of vaginal delivery and are able to bear the costs, women in the poorest households are underutilizing CS because they are too poor to pay [14, 15]. Like most sub-Saharan African countries, Tanzania did not meet the Millennium Development Goal (MDG) in reducing maternal mortality [16] and 556 maternal deaths per 100,000 live births occurred in 2015 [17]. In the midst of the Sustainable Development Goal of reducing maternal mortality ratio to less than 70 per 100,000 live births by the year 2030 [18], most mothers in Tanzania still die because of direct obstetric causes, which is associated with far distance from health facility mainly from hospital, which indirectly indicates women living in rural settings are more affected as CS rate has been found to also decrease with distance [19, 20]. Evidence show that the national CS coverage was 6% in 2015–2016 [17, 19, 21]. However, the utilization coverage varied from 4% in rural to 12% among urban residents [21]. Despite the importance of inequality analysis of CS in producing evidence on the state of within-country variations in CS across different population subgroups, in Tanzania, there is little information on this issue using rigorous and well-established approaches. High-quality evidence is really needed to help close the gap between various population groups in the country, and to eventually ensure reasonable access of the service to all who need it. In this study, we aimed to comprehensively investigate both the magnitude and time trends of socio-economic, rural-urban and subnational inequalities in the use of CS in Tanzania using five waves of the Tanzania Demographic and Health Survey (TDHS).

## Materials and methods

### Brief overview of the study setting

Tanzania is a country that has a a population of about 55 million as of 2016 and it is situated in the Eastern part of Africa [22]. The country has several climatic and topographic condition, which range from the hot and humid coastal lowlands of the Indian Ocean shoreline to the high inland mountain and lake region of the northern border, making it the home to different flora and fauna life [23]. Over the last decade, Tanzania has recorded relatively high economic growth, averaging 6–7% annually. There is evidence of an increase in real gross domestic product (GDP) growth from 6.8% in 2017 to 7% in 2018. Although the country managed to reduce its rate of poverty, the same success has not been repeated with respect to reducing the absolute number of poor people in the country due to high rate of population growth. Efforts by the government to boost coverage of social services like education, health, and water have been hampered by their declining quality as the size of the population \does not correspond to the supply of the services [22].

Tanzania has a Human Development Index (HDI) value of 0.528 and ranks 159 out of the 189 countries and territories in 2018. The human development report positioned the country in the low human development category, an indication of poor performance in the three important dimensions of the human development, namely life expectancy, decent standard of living and accessibility to knowledge and learning [24].

In Tanzania, the probability of children under five dying before celebrating their fifth birthday is 53 deaths per 1000 live births. While neonatal mortality remained unchanged, Tanzania had seen a fall in the burden of post neonatal mortality rates, child mortality rates, infant mortality and under-five mortality rates [25]. Individuals aged 15 to 60 have a probability of dying of 299 and 222 deaths per 1000 population respectively. Expenditure on health has a share of 5.6% of the total Gross Domestic Product (GDP) [26].

Tanzania’s health system follows a pyramidal structure, from village dispensaries and community-based activities at the base (under the responsibility of local government authorities), to ward, district, and regional level hospitals and finally referral and national hospitals at the summit. The government runs four health insurance schemes alongside multiple private options, but the vast majority of the population remains uninsured, leading to significant inequities in access to care. Tanzania’s 4th Health Sector Strategic Plan (2015–2020) provides for a new health financing strategy aimed at helping the country achieve universal health coverage, by addressing this complex and fractured health insurance market [27].

### Data sources

Data from the 1996 to 2015 TDHS, which are publicly available via Measure DHS were used in this study. TDHS are nationally representative household surveys with a strong focus on maternal and child health issues such as fertility levels and preferences, marriage, sexual activity, awareness and use of family planning methods, breastfeeding practices and use of maternal healthcare services [17]. DHSs serve as important sources of data for monitoring population health indicators and vital statistics in low- and middle-income countries and known by their design, which are highly comparable among different settings and over time. The sample design, selection and methodology of survey approach in each round were similar and has been available elsewhere [17].

### Selection of variables

Inequality in CS delivery 5 years preceding the surveys was measured for four equity stratifiers (economic status, education, place of residence and region). In this study, we refer to CS as primary variable and we do not use the word ‘outcome’ as we did not run any regression-based model. CS was measured as proportion of births that occurred via CS in the 5 years prior to the surveys.

The World Health Organization (WHO) has defined equity stratifiers, also known as dimensions of inequality, as subpopulations that are used to disaggregate health indicators [9]. According to the WHO, a health inequality should be analyzed and interpreted using all dimensions of inequality as far as the available dimensions are relevant for the health indicator of interest, as well as data is available for each category of the subpopulations. In health disparity literature, big attention has been given to health inequality by economic status. However, the WHO recommends other dimensions as well such as place of residence, race or ethnicity, occupation, gender, religion, education, and social capital or resources.

In the present study, we employed four dimensions of inequality to analyze CS inequality: economic status, education status, residence and subnational regions. Our selection of the equity stratifiers was based on the fact that they are relevant to CS and data on CS were also available for each of them. Economic status was approximated through a wealth index in the DHS computed using easy-to-collect data on household assets and ownerships such as televisions and bicycles; materials used for housing construction; and types of water access and sanitation facilities, following the methodology explained elsewhere [28] and was categorized into poorest, poorer, middle, richer and richest. Wealth index was computed for each of the four surveys conducted in Tanzania using principal component analysis (PCA) [29]. The wealth index variable used here is comparable across the survey years. In large household surveys like DHS where data on income cannot be collected, wealth index has been used as a proxy for household income and or expenditure measures [30]. The wealth index is a summary measure that reflects a household’s total economic well-being and allows for the identification of problems particular to the poor, such as unequal access to health care, as well as those particular to the wealthy, such as elevated risk of contracting HIV infection [28]. Maternal educational status was classified as no-education, primary education, and secondary education, place of residence as rural vs. urban and sub-national regions categorized into 30 regions.

### Data analysis

The latest version of the WHO’s HEAT software was adopted for the analysis [31]. In the software, CS delivery were analyzed and disaggregated by the four equity stratifiers-economic status, education, place of residence and region and were presented through the four of the 15 commonly used summary measures of health inequality [29]. In addition to disaggregation, we computed summary measures of inequality. Out of the 15 summary measures available in the software, we chose to use four, namely Difference (D), Ratio (R), Slope Index of Inequality (SII) and Relative Index of Inequality (RII) due to their wider application in health care inequality studies [9, 32].

Both simple and complex summary measures were calculated for each equity stratifier to better understand inequality involved in the utilization of CS delivery [9]. For the economic status and education dimensions of inequality, Difference, Ratio, SII and RII were used. For place of residence, Difference and Ratio were calculated. Difference and Ratio are simple measures of health inequality, whereas the SII, and RII are complex measures [29]). While simple measures of health inequality are suitable for pairwise comparison of a health indicator of interest, they do not account for the subpopulations in the middle when applied to an equity stratifier with more than two categories, such as wealth index. This issue is avoided by the adoption of complex measures, whereby estimates are based on the sizes of all categories of a particular dimension of inequality [9, 29].

As step-by-step procedure for the calculation of each summary measure included in the health equity database are discussed in detail in the HEAT software technical notes [29] and the WHO handbook on the health inequality monitoring [9]. With economic status and education dimensions of inequality, Difference was calculated as CS delivery in the richest group minus in the poorest group, and CS delivery utilization among the group that has acquired at least secondary education minus the uneducated group. Similarly, for place of residence, Difference pertains to what exists between urban and rural populations. Finally, with the sub-national regions, Difference relates to the Difference between regions with the highest and the lowest CS coverage.

R is calculated as the ratio of two subgroups: *R* = Y_high_ / Y_low_. For place of residence, Y_high_ and Y_low_ are urban and rural residents respectively. Whereas in educational status, Y_high_ and Y_low_ refers to respectively the most advantaged subgroups which are secondary schools and above and the most disadvantaged are subgroups with no education groups. In case of economic status, Y_high_ and Y_low_ refers to the most advantaged subgroups which are the richest quintile and the most disadvantaged subgroups which are the poorest quintile respectively.

Finally, SII and RII were calculated through a generalized linear model with a logit link. Their computation was restricted to ordered dimensions (education and economic status) and requires ranking of a weighted sample in order from the most disadvantaged (rank 0) to the most advantaged (rank 1) subgroups. The poorest and uneducated individuals were considered the most disadvantaged, but those that have completed secondary education and the richest subgroups were deemed most advantaged. Then, CS delivery was predicted for those at the two extremes and the difference in the predicted value between rank 1 and rank 0 produces SII. The RII was computed by dividing the predicted cesarean section delivery coverage for rank 1 by that of rank 0.

Owing to the complex sampling structure of the DHS datasets, our analysis took this complexity into account in order to generate findings that are not biased as well as are representative. That is, the survey specifications were considered during analysis to redress problems introduced because of the sampling process and to generate reliable findings.

As a measure of statistical significance, 95% Confidence Intervals (CI) were computed around point estimates. While interpreting inequality existence, Difference and SII lower and upper bounds of CI shall not entail zero. R and RII inequality exist if CIs do not involve one. In the case of inequality trend interpretation, CIs of the summary measures for different survey years shall not overlap to conclude a change in inequality over time.

### Ethical consideration

We did the analyses using publicly available DHS dataset. Because the ethical clearance was approved by the institution that commissioned, funded and managed the overall DHS program, further ethical clearance was not required. Informed consent from the participants prior to survey was obtained in the course of the survey. ICF international and the ethical review Board (IRB) of Tanzania also ensured that the protocols are in compliance with the U.S. Department of Health and Human Services regulations for the protection of human subjects.

## Results

In this study, a total of 37,145 participants were involved in all five surveys. Of them, 29,220 (78.6%), 9465 (25.4%) and 8461 (22.7%) were rural residents, non-educated and among wealth quintile 1 subgroups, respectively. The national average coverage of birth by CS was 2.1, 2.9, 3.2, 4.4 and 5.9% in 1996, 1999, 2004, 2010 and 2015 respectively.

Table 1 shows variations in the utilization of CS birth across socio-economic, urban-rural and regional subgroups in Tanzania from 1996 to 2015. For instance, in the first (1999) and last (2015) surveys, the coverage among the poorest categories was 1% (95% CI: 0.55, 1.90) and 2.4% (95% CI: 1.75, 3.29) respectively, while 5.2% (95% CI: 3.96, 6.90) and 15.8% (95% CI: 13.17, 18.85) were recorded among richest subpopulations respectively. Even if the extent was varied, in all subgroups of wealth index increment pattern were observed if we take and seen the first and the last surveys. For instance, among the poorest subgroups, the increment was, based on the value of the point estimates, by 1.38 percentage points (pp) over the last 19 years whereas by 10.5 pp. among the richest subgroups within same period of time (Table 1).

**Table 1:** Coverage of caesarean section birth across socioeconomic subgroups and geographic locations in Tanzania from 1996 to 2015

Dimension of inequalities	Year									
	1996		1999		2004		2010		2015	
	%(95%CI)	Popn	%(95%CI)	Popn	%(95%CI)	Popn	%(95%CI)	Popn	%(95%CI)	Popn
Economic status										
Quintile 1 (poorest)	1.02 (0.55, 1.90)	1587	0.78 (0.29, 2.04)	747	0.97 (0.58, 1.61)	1973	1.76 (1.14, 2.70)	1728	2.40 (1.75, 3.29)	2426
Quintile 2	1.30 (0.80, 2.11)	1306	1.41 (0.6, 3.32)	692	2.25 (1.57, 3.20)	1856	2.32 (1.66, 3.23)	1928	2.59 (1.72, 3.89)	2134
Quintile 3	2.12 (1.38, 3.23)	1426	2.36 (0.96, 5.68)	700	2.99 (2.08, 4.29)	1865	3.46 (2.41, 4.93)	1836	4.20 (3.09, 5.69)	1928
Quintile 4	1.47 (0.88, 2.43)	1408	4.03 (1.96, 8.10)	575	2.81 (1.96, 4.00)	1680	5.69 (4.38, 7.36)	1525	7.14 (5.69, 8.93)	1887
Quintile 5 (richest)	5.24 (3.96, 6.90)	1187	7.27 (4.62, 11.27)	565	8.68 (6.51, 11.47)	1347	12.00 (9.50, 15.06)	1156	15.80 (13.17, 18.85)	1674
Education										
No education	1.17 (0.73, 1.88)	2048	0.91 (0.38, 2.15)	906	1.07 (0.69, 1.65)	2318	2.18 (1.44, 3.30)	2090	2.12 (1.47, 3.04)	2103
Primary school	2.34 (1.84, 2.96)	4631	3.69 (2.41, 5.61)	2258	3.45 (2.87, 4.13)	6019	4.36 (3.69, 5.13)	5569	5.06 (4.37, 5.84)	6516
Secondary school +	5.96 (3.62, 9.68)	235	4.21 (1.89, 9.10)	116	12.57(7.27, 20.85)	386	14.70(10.74, 19.79)	516	15.37 (12.32, 19.02)	1431
Residence										
Rural	1.69 (1.33, 2.15)	5680	2.05 (1.28, 3.26)	2667	2.11 (1.73, 2.59)	7033	3.15 (2.62, 3.79)	6516	3.71 (3.15, 4.37)	7324
Urban	4.07 (2.99, 5.51)	1235	6.83 (3.43, 13.13)	614	7.81 (5.84, 10.36)	1691	9.57 (7.48, 12.18)	1659	11.82 (9.67, 14.37)	2726
Region										
01 dodoma	1.80 (0.65, 4.86)	311	9.71 (3.69, 23.18)	144	0.64 (0.16, 2.52)	412	2.75 (1.53, 4.89)	462	5.57 (3.13, 9.71)	424
02 arusha	3.89 (2.14, 6.98)	547	3.61 (0.61, 18.57)	430	3.70 (1.35, 9.73)	287	5.08 (2.81, 9.01)	299	8.22 (4.47, 14.62)	349
03 kilimanjaro	4.59 (3.22, 6.50)	280	2.74 (0.76, 9.28)	109	7.18 (3.58, 13.88)	209	11.03 (7.13, 16.70)	190	9.69 (5.62, 16.21)	168
04 tanga	1.91 (0.94, 3.85)	364	0.59 (0.20, 1.71)	138	2.66 (1.22, 5.67)	354	4.71 (2.26, 9.54)	340	8.74 (5.55, 13.50)	416
05 morogoro	1.65 (0.58, 4.56)	327	9.13 (4.76, 16.81)	134	8.93 (3.49, 20.96)	349	5.80 (3.14, 10.46)	355	6.20 (3.01, 12.31)	440
06 coast	2.74 (0.87, 8.25)	104	0.56 (0.06, 4.82)	113	1.60 (0.48, 5.23)	191	5.76 (2.66, 11.99)	205	2.95 (1.17, 7.22)	203
07 dar es salaam	4.70 (3.30, 6.65)	376	8.90 (5.71, 13.63)	140	7.72 (4.48, 12.98)	428	13.39 (8.72, 20.01)	402	17.02 (11.69, 24.11)	771
08 lindi	0.90 (0.22, 3.60)	129	3.82 (1.27, 10.92)	68	4.72 (2.94, 7.49)	146	3.37 (1.46, 7.56)	152	6.58 (3.20, 13.03)	176
09 mtwara	2.06 (0.93, 4.50)	234	4.69 (1.04, 18.63)	88	2.41 (0.95, 5.95)	246	5.82 (3.52, 9.47)	237	10.34 (6.10, 16.99)	215
10 ruvuma	2.87 (1.74, 4.71)	250	0 (0, 0)	88	5.04 (2.85, 8.76)	243	8.22 (4.80, 13.72)	248	9.99 (6.36, 15.33)	248
11 iringa	1.68 (0.56, 4.91)	354	2.53 (0.56, 10.56)	106	6.60 (4.23, 10.17)	294	9.73 (5.55, 16.52)	323	11.94 (8.14, 17.18)	162
12 mbeya	3.31 (1.38, 7.72)	362	5.00 (2.34, 10.35)	176	1.53 (1.08, 2.17)	662	2.52 (0.76, 8.00)	515	6.85 (4.41, 10.50)	559
13 singida	2.22 (1.21, 4.05)	258	0 (0, 0)	100	3.11 (1.68, 5.69)	302	2.79 (1.16, 6.54)	298	4.83 (2.82, 8.18)	334
14 tabora	3.33 (1.05, 10.04)	170	0 (0, 0)	111	2.28 (1.23, 4.17)	508	4.84 (2.57, 8.92)	453	2.68 (1.49, 4.79)	712
15 rukwa	0.56 (0.16, 1.98)	241	1.02 (0.08, 11.08)	85	0.46 (0.11, 1.90)	326	5.18 (3.11, 8.49)	288	3.98 (2.47, 6.35)	276
16 kigoma	1.11 (0.38, 3.19)	342	3.67 (1.81, 7.31)	99	1.90 (0.57, 6.07)	484	2.00 (0.84, 4.68)	432	3.95 (2.24, 6.87)	513
17 shinyanga	1.15 (0.58, 2.24)	634	0 (0, 0)	304	1.45 (1.17, 1.80)	919	2.14 (1.09, 4.16)	836	3.64 (1.80, 7.22)	467
18 kagera	1.21 (0.48, 3.05)	539	3.17 (0.80, 11.67)	221	3.79 (2.11, 6.71)	573	2.71 (1.26, 5.72)	511	2.92 (1.42, 5.90)	533
19 mwanza	1.27 (0.26, 5.80)	580	0.56 (0.14, 2.25)	320	4.58 (2.85, 7.30)	898	3.50 (1.96, 6.18)	791	3.24 (1.48, 6.95)	736
20 mara	0.66 (0.09, 4.27)	281	2.44 (0.52, 10.72)	212	0.63 (0.20, 1.94)	395	0.57 (0.14, 2.25)	406	4.00 (2.37, 6.66)	495
21 pemba	0.62 (0.09, 4.05)	98	0.94 (0.14, 5.76)	41	2.35 (1.02, 5.31)	269	1.39 (0.55, 3.47)	199	4.31 (2.51, 7.31)	351
22 rest Zanzibar	1.72 (0.65, 4.47)	123	1.24 (0.56, 2.72)	44	0.52 (0.11, 2.31)	33	3.66 (1.76, 7.46)	35	13.72 (8.35, 21.74)	131
23zanzibar south	NA	NA	NA	NA	1.81 (0.76, 4.26)	18	5.65 (3.11, 10.07)	18	0.76 (0.24, 2.34)	138
24 town west	NA	NA	NA	NA	3.08 (1.46, 6.37)	81	7.80 (5.28, 11.38)	77	1.07 (0.42, 2.70)	496
25 pemba north	NA	NA	NA	NA	1.38 (0.62, 3.05)	44	0.55 (0.14, 2.13)	45	2.08 (1.01, 4.25)	463
26 pemba south	NA	NA	NA	NA	1.26 (0.63, 2.51)	41	4.15 (2.01, 8.40)	44	4.16 (2.42, 7.03)	45
27 kusini unguja	NA	NA	NA	NA	NA	NA	NA	NA	3.94 (2.04, 7.48)	25
28 mjini magharibi	NA	NA	NA	NA	NA	NA	NA	NA	11.52 (8.82, 14.90)	94
29 kaskazini pemba	NA	NA	NA	NA	NA	NA	NA	NA	2.31 (1.29, 4.11)	52
30 kusini pemba	NA	NA	NA	NA	NA	NA	NA	NA	1.91 (0.79, 4.56)	45
Total measles	2.12		2.94		3.22		4.45		5.91	

N.B Regions from 21 to 26 listed above are not for last survey, for survey 2015 are 21 manyara, 22 njombe, 23 katavi, 24 simiyu, 25 geita, and 26 kaskazini unguja, NA = not applicable

Access to birth by CS across educational subgroups also varied in all of the survey rounds. Although constant pattern was generally observed, incremental changes were found when we compared the first and last surveys. For instance, the coverage was increased, based on the point estimates, by 0.95 percentage points, 2.7 percentage points and 9.4 percentage points among non-educated, those who attended primary school secondary or higher school, respectively over the last 19 years (Table 1).

CS utilization variation was also seen among rural and urban residents in all survey rounds in Tanzania with high coverage among the urban residents as compared to their rural counterparts. The pattern was constant in both subgroups except increasing pattern between the first and the last rounds of surveys. The coverage among rural residents was increased by 2 percentage points, whereas by 7.8 percentage points among urban residents over the last 19 years. There were also utilization differences among different regions within the country. For more details see Table 1.

### Extents and trends of CS coverage inequality

The finding from the current study shows that except with Ratio measure in 1999, there were substantial absolute and relative wealth-driven inequalities in access to birth by CS by all four measures from 1996 to 2015. For instance, the Ratio measure (6.55, 95% CI, 4.18, 8.93) in the 2015 survey indicates that CS service among richest women was nearly 4 to 9 times higher as compared to poorest women. The over time change of the wealth driven CS inequality shows that the disparity had widened between the first and the last surveys though the disparity generally remained unchanged between the earlier rounds of surveys.

With the exception in 1999, by Difference, Ratio and RII, we found significant education related disparities in access to birth by CS in all other survey periods. Similar to the wealth related inequality, educational inequality worsened over time, especially between the first and the last surveys. The figure of RII (13.71, 95% CI; 9.04, 18.38) and SII (16.04, 95% CI; 13.58, 18.49) measures in 2015 survey indicate, substantial relative and absolute education-based inequalities in access to birth by CS service respectively, with better uptake among educated women as compared to non-educated counterparts.

We also examined urban-rural disparity with the simple measures. The findings show that except absence of inequality in 1999 by Ratio measure, substantial absolute and relative place of residence inequality was observed in all survey rounds in Tanzania with constant disparity pattern over the last 2 decades by the ratio measure. However, the Difference measure showed that CS disparity widened between the first and the last surveys. For example, the disparity increased by nearly 6 percentage points between the first and the last rounds of the TDHS. We also recorded substantial absolute regional disparity in access to birth by CS. The Difference measure (16.3, 95% CI; 10.02, 22.48) in recent survey (2015) indicates CS was higher by 16.3 percentage point among women living in regions such as Dar es salaam as compared to women living Zanzibar south region (Table 2). However, there was no relative regional inequality.

**Table 2:** Extents and trends of socioeconomic, urban-rural and regional disparities in caesarean section birth in Tanzania from 1996 to 2015

Dimension	Year					
		1996	1999	2004	2010	2015
	Measure	%(95%CI)	%(95%CI)	%(95%CI)	%(95%CI)	%(95%CI)
Wealth	D	4.21 (2.64, 5.79)	6.49 (3.20, 9.79)	7.70 (5.21, 10.20)	10.24 (7.38, 13.10)	13.39 (10.46, 16.32)
	R	5.11 (1.66, 8.55)	9.32 (−0.47, 19.11)	8.90 (3.78, 14.02)	6.81 (3.50, 10.12)	6.55 (4.18, 8.93)
	RII	7.37 (2.77, 11.97)	16.21 (2.80, 29.61)	10.96 (5.87, 16.05)	12.52 (7.38, 17.66)	15.55 (10.44, 20.66)
	SII	4.21 (2.74, 5.68)	8.17 (5.34, 11.01)	7.66 (5.97, 9.34)	11.13 (9.09, 13.16)	15.80 (13.70, 17.91)
Education	D	4.79 (1.81, 7.76)	3.30 (−0.06, 6.66)	11.49 (4.85, 18.14)	12.51 (7.94, 17.09)	13.25 (9.83, 16.67)
	R	5.07 (1.63, 8.51)	4.60 (−0.68, 9.89)	11.72 (3.74, 19.70)	6.72 (3.27, 10.17)	7.24 (4.19, 10.29)
	RII	5.77 (1.24, 10.30)	8.84 (−0.17, 17.86)	20.74 (8.05, 33.42)	10.75 (5.49, 16.02)	13.71 (9.04, 18.38)
	SII	3.87 (1.91, 5.84)	6.77 (3.00, 10.54)	10.92 (8.04, 13.81)	11.20 (8.46, 13.95)	16.04 (13.58, 18.49)
Residence	D	2.37 (1.07, 3.67)	4.78 (0.14, 9.43)	5.69 (3.42, 7.95)	6.42 (4.01, 8.82)	8.10 (5.68, 10.52)
	R	2.40 (1.47, 3.32)	3.33 (0.63, 6.03)	3.68 (2.40, 4.97)	3.03 (2.10, 3.96)	3.18 (2.36, 3.99)
Region	D	4.14 (2.35, 5.92)	9.71 (0.78, 18.64)	8.46 (0.38, 16.54)	12.83 (7.21, 18.45)	16.25(10.02, 22.48)
	R	8.31 (−2.49, 19.11)	NA	19.07 (−12.74, 50.89)	23.96 (−9.76, 57.69)	22.19 (−3.99, 48.37)

*D* Difference, *R* Ratio, *RII* Relative Index of Inequality, *SII* Slope Index of Inequality, *NA* Not applicable

## Discussion

This study explored over time trends in socioeconomic and geographic disparities in access to birth by CS over the course of roughly two decades in Tanzania. Overall the findings of this study indicate considerable inequalities in the service uptake of CS between the rich and the poor, educated and non-educated, richest/rich and poorest/poor, urban and rural, and between regions, with varying over time trends.

Consistent with previous studies [5, 33–35], we found a disproportionately higher CS birth service uptake among richest/rich women as compared to poorest/poor women. Poor access to delivery by caesarean section remains mainly an issue among the economically and the socially deprived, which has serious implications for reducing maternal mortality and morbidity and perinatal and neonatal mortality and morbidity in the country [36]. Because of unaffordability of cost for CS service sometimes women are not able to deliver via CS (37) and hope for positive birth outcome even in case of prolonged labor since cost of CS is beyond their capability (38) as supported by previous studies that paying capability to CS service is one of the determinant for access to birth by CS [37]. Further, this corroborates previous study in Ethiopia, where women belonged to the richest household wealth quintile had higher odd of CS use than women in the poorest wealth category, indicating the potential impact of health care costs to access to CS delivery [15]. Evidence suggests that women requiring CS are particularly vulnerable to the problem of “medical detention” for inability to pay medical bill [38], indicating that poor women could not be able to attend CS services and thus contributing to a persistent poor-rich CS disparity. A developing country-based evidence showed that financial constraints have been cited as a reason to refusal to CS delivery [37].

We found better CS service uptake among educated women than non-educated women as confirmed in prior studies (34, 37. In Africa, by prospecting vaginal delivery will occur, poor birth outcome finally happened even with CS performed [39], because of delay in getting CS delivery timely [40]. The possible mechanism through which higher level of education increases the odd of CS delivery could be that, more educated women are likely to develop decision making power, which in turn, may rise the chance that women could get CS service [41]. Besides, evidence showed better health awareness could be seen among educated mothers as compared to their non-educated counterparts and this differential of health awareness could lead to disparity in CS use [35, 41, 42]. Available literature disclosed that health literacy has been shown to improve health equity [42] and the fact that health literacy is linked with educational attainment could lead us to the assertion that highly educated women are more likely to access health service such as CS delivery than non-educated counterparts. Incongruent to prior work, education has been shown to not affect the decision to deliver via CS [43, 44] and the impact of education on whether to receive health care services can sometimes be nullified by cultural and religious beliefs.

Comparable with previous study in Africa [14, 45]. CS service were more concentrated among urban residents and certain regions. Poor preparation in terms of equipment and related supplies as well as trained professionals such as Doctors and Anesthetist and availability of low density health facilities among rural public health facilities explained this as supported by prior study in Tanzania [46]. In terms of sub-national regions, we found inequality in the coverage of CS with disproportionately higher uptake in some regions such as Dar Es Salaam. Noticeable geographic variation in the level of health facility readiness in the country had been established in prior study [18] and this variation in the capacity to provide CS can in turn lead to skewed distribution of CS service with some parts of the country reporting higher coverage. The smallest proportion of facilities meeting readiness criteria was found in the Southern (14%) and Western zones (19%), where only few proportions of women deliver through CS [18]. On the other hand, Lake, Northern and Central zones are equipped with facilities with better level of readiness required to provide CS. This amounts to the fact that the observed glaring disparity in the capacity of health facilities across the subnational regions could result in higher clustering of preventable maternal deaths in the underperforming regions due to lower coverage of CS. Maswanya and al. found out that policy restrictions, lack of supplies and professional development, and operating under lowly developed referral services were mentioned as the some of the drivers for low and inconsistent use of obstetric care services in western zones of Tanzania [47].

### Strengths and limitations of the study

Investigating disparities using simple and complex as well as relative and absolute measures made the study more valuable due to its importance in looking at the disparities from different outlook in addition to its importance in overcoming one method by the strength of other methods could be major strength of the study. Second, examining inequalities from four dimensions can hugely support in highlighting where government and other stakeholders need to focus to build up their efforts towards realization of the equity-oriented SDG targets in relation to maternal health. Third, the study used the high-quality data available through the well-established WHO health equity monitor database contributing to the quality of the conclusions drawn from the study. Finally, owing to the complex sampling structure of the DHS datasets, our analysis took this complexity into account in order to generate findings that are not biased as well as are representative. That is, the survey specifications were considered during analysis to redress problems introduced because of the sampling process and to generate reliable findings. However, the study has some limitations. Our analysis could not provide an in-depth assessment of problems that led to the observed CS disparities, making it challenging for decision makers to put in place targeted interventions. Future studies need to apply a decomposition method to better appreciate the level of influences of common problems on the observed CS disparity. Also, the study presented inequality of the service at population level, and studies are also required to compare with CS disparities at health facility level. Finally, since the data for CS was collected for 5 years prior to the surveys, there could be the possibility of recall bias. However, it is unlikely that mothers may forget remembering whether they gave birth via CS 5 years ahead of the surveys and that it seriously impacted our conclusions contained in the paper. Also, it is possible that some respondents might not respond correctly on the mode of delivery and this might have affected our findings by decreasing the sample size.

## Conclusions

What is novel in this paper is that, the analysis was carried out in accordance with the WHO recommendation for equity studies; simple and complex, as well as relative and absolute summary measures were calculated in order to measure the disparity from different perspectives and viewpoints. This practice mitigates the problem of false positive and negative findings as different measures are likely to capture different version of the same disparity. The finding showed that over the last 19 years, women who were uneducated, poorest/poorer, lived in rural settings and from regions such as Zanzibar South, appeared to be less likely to utilize CS services in Tanzania. More work needs to be done to ensure that all subpopulations that require medically necessary CS are able to access the service in order to avoid preventable maternal and infant deaths. It is also important to discourage unjustified use of CS among certain subgroups to reduce deaths associated with unnecessary CS delivery. The findings are all policy relevant; interventions that aim to redress the observed inequality in the coverage of CS should be targeted and context specific. The subpopulations that are lagging behind require different interventions from those who are using the service above the optimal recommended rate. Since understanding the drivers of CS inequality are not captured in the current paper, we strongly recommend the conduct of decomposition analysis to document factors that operate behind the disparity. Such information would be used by policy makers to ensure justified use of the service across all subpopulations in the country. Finally, our findings support the assertion that CS service can be improved by redressing the social and geographic variations; by increasing access to secondary education, by creating job opportunity for poor citizens and paying particular attention to some regions that are performing poorly in terms of increasing CS compared to other regions in the country.

## Data Availability

The datasets generated and/or analyzed during the current study are available in the WHO’s HEAT version 3.1 [https://www.who.int/gho/health_equity/assessment_toolkit/en/].

## Abbreviations

CS: Caesarean section
D: Difference
HEAT: Health equity assessment toolkit
PPS: Probability proportional to size
R: Ratio
RII: Relative index of inequality
SDG: Sustainable development goal
SII: Slope index of inequality
TDHS: Tanzania demographic and health survey
WHO: World health organization
